# Management of calcific myonecrosis with a sinus tract: A case report

**DOI:** 10.1097/MD.0000000000012517

**Published:** 2018-09-21

**Authors:** Bi O Jeong, Duke Whan Chung, Jong Hun Baek

**Affiliations:** Department of Orthopaedic Surgery, Kyung Hee University Hospital, College of medicine, Kyung Hee University, Seoul, Korea.

**Keywords:** calcific myonecrosis, compartment syndrome, dystrophic calcification, surgical treatment

## Abstract

**Rationale:**

Calcific myonecrosis is a very rare late sequela that occurs in patients who have had trauma accompanied by vascular compromise, in which a single muscle or entire muscles in a compartment undergoes necrosis and form a calcified mass. It is mostly a benign entity, but some cases cause bone destruction and form non-healing chronic sinuses. In such cases, wound management becomes difficult and there is a potential risk of secondary infection.

**Patient concerns:**

A 60-year-old male was referred for evaluation of a pain, erythematous changes, and draining sinus of the anterolateral aspect of his left leg. He had an open reduction and internal fixation as well as a stent insertion in the femoral artery owing to a distal femur fracture and femoral artery rupture.

**Diagnoses:**

A thick fluid with a chalk-like material was discharged through the shiny skin via the sinus. The radiographs of the left leg showed a large, fusiform-shaped, radiopaque soft tissue mass in the space between the tibia and fibula. We performed an incisional biopsy to differentiated soft tissue sarcoma and malignant cells were found. Pathologic evaluation revealed acute and chronic inflammation with dystrophic calcification. These findings led to the diagnosis of calcific myonecrosis.

**Interventions:**

We performed an extensive debridement of the anterior and deep posterior compartments to ensure definitive treatment. Upon performing extensive debridement, we inserted a drain tube and performed primary closure.

**Outcomes:**

The fluid continued to be discharged through the drain even after the surgery; delayed wound healing occurred 4 weeks following the surgery, and there was no recurrence at follow-up conducted 2 years later.

**Lessons:**

Calcific myonecrosis is mostly a benign entity, but some cases of calcific myonecrosis cause bone destruction and form non-healing chronic sinuses. In such cases, surgical treatment is required, during which the necrotic tissue and calcific material must be extensively debrided and drained.

## 1 Introduction

Calcific myonecrosis is a disease in which a single muscle or an entire muscle in a compartment undergoes necrosis and form a calcified mass. It is a rare late sequela that occurs in patients who have had trauma accompanied by vascular compromise, such as in compartment syndrome or subclinical compartment syndrome. The condition mostly occurs in the anterior compartment of the lower limb and, in rare cases, in the upper extremity.^[1]^ The condition usually manifests as a firm and enlarging mass with or without pain several decades following the initial trauma, sometimes accompanied by multiple discharge sinuses or infections.^[2]^

Although the pathophysiological mechanism of calcific myonecrosis is not well understood, it is thought that neurovascular compromise leads to muscle necrosis, fibrosis, and subsequent liquefactive cystic degeneration, as well as calcification of the fibrous tissue.^[3]^ However, the reason for the delayed calcification is unclear.

Treatment of calcific myonecrosis can vary depending on the symptoms, from conservative management to a complete surgical debridement.^[4]^ Surgical treatment is a definitive treatment option, but is associated with a high risk of postoperative complications, such as infection or chronic sinus formation.^[2]^ In case of sinus formation, wound management becomes difficult and there is a potential risk of secondary infection. We report a case of calcific myonecrosis in a patient who had leg trauma 18 years before the formation of enlarging mass with a sinus tract. The clinical data and images were obtained with informed consent of the patient including consent to use the photographs in this report, according to the Declaration of Helsinki.

## 2 Case report

A 60-year-old male who had a history of liver cirrhosis was referred for evaluation of a pain, swelling, and erythematous changes of the anterolateral aspect of his left leg. His symptoms started a month ago without any specific trauma. He had a history of open reduction and internal fixation performed for a tibial plateau fracture that occurred when his left knee was pinned under a tree 18 years ago. He had sciatic nerve injury at the time of the fracture. He had an open reduction and internal fixation as well as a stent insertion in the femoral artery 8 years after the initial trauma due to a distal femur fracture and femoral artery rupture. At that time, a widespread radiopaque mass was observed on plain radiographs of left leg, and obstruction of popliteal artery with abundant collateral circulation was observed on angiography. The patient had no difficulty with his daily life functions until the aforementioned symptoms appeared, except for limited motion in the ankle and toes and sensory reduction in his foot owing to the sciatic nerve injury.

After admission, the patient maintained with a long leg splint. Two weeks after the admission, the erythematous changes in the left leg localized to the anterior aspect in the middle of the leg and formed a fluid-filled, erythematous mass. A draining sinus had developed on the erythematous mass spontaneously and a thick fluid with a chalk-like material was discharged through the shiny skin via the sinus. The range of motion of the knee was 0 degrees to 100 degrees of flexion; ankle dorsiflexion was 0 degrees, ankle plantar flexion was 5 degrees, and all of the toes had clawing deformity. As for the motor strength of the ankle, the dorsiflexion was grade 0 and plantar flexion was grade 3. He had no sensation in the first web space and on the medial aspect of the foot, and had sensory reduction in the lateral, dorsal, and plantar aspects of the foot, in order of decreasing sensation.

**Figure 1:**
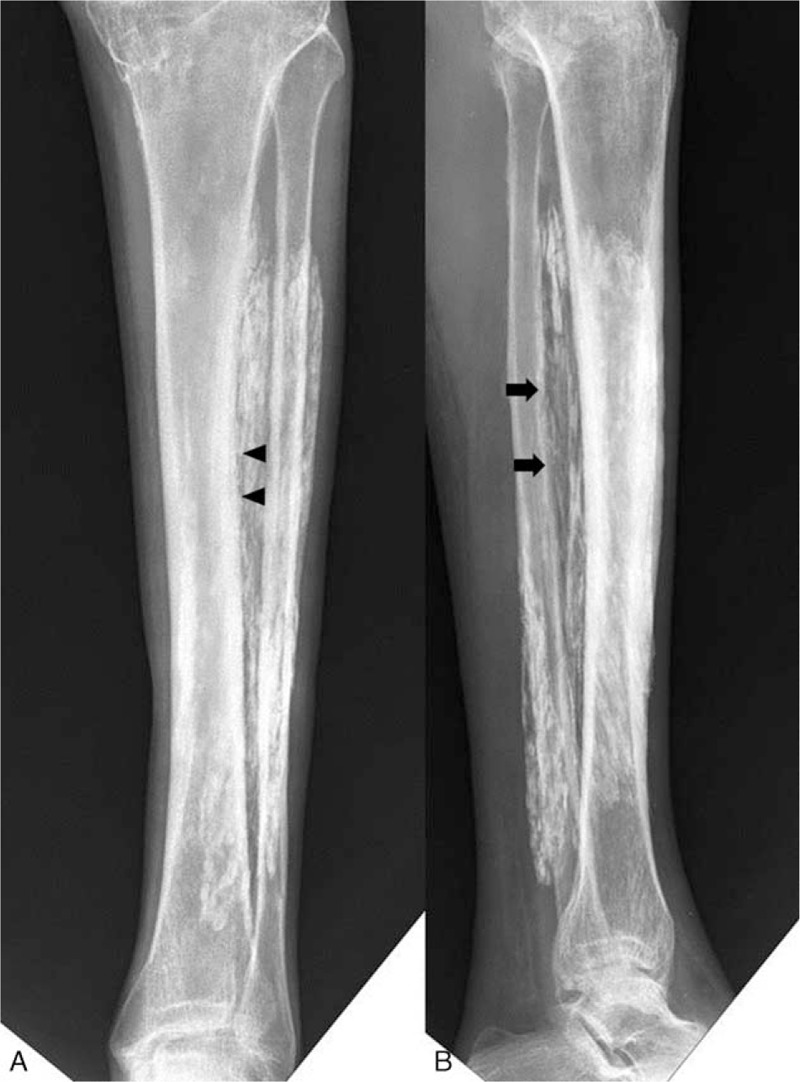
At 18 years after the initial trauma, radiographs showing an extensive radiopaque mass with plaque-like and amorphous calcifications. Anteroposterior view (A) showing sclerotic change with erosion in the lateral cortex of the middle third of the tibia (arrow head). Also lateral view (B) showing sclerotic with erosion in the anterior cortex of the middle third of the fibula (arrow).

**Figure 2:**
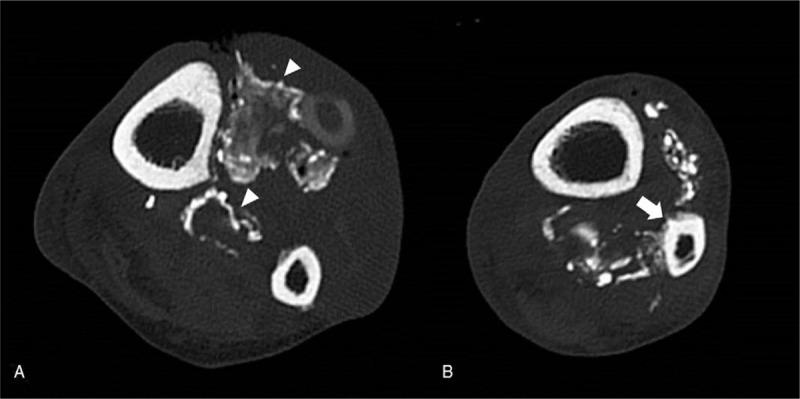
Axial computed tomography scan (A) showing a predominantly peripherally distributed calcified mass replacing the anterior and deep posterior compartment muscles of the leg (arrow head). Also, it (B) shows sclerotic changes and erosion of the medial and posterior cortices of the fibula (arrow).

The radiographs of the left leg taken 10 years ago (8 years after the initial trauma) showed a large, fusiform-shaped, radiopaque soft tissue mass in the space between the tibia and fibula that spanned from 5 cm below the proximal tibio-fibular articulation to just above the distal tibio-fibular articulation. The medial cortex of the middle third of the fibula and the posterior cortex of the distal third were sclerotic. Ten years later (18 years after the initial trauma), the patient's radiographs showed a large, fusiform-shaped soft tissue mass with extensive plaque-like and amorphous calcifications, similar to those seen in the radiographs taken 10 years earlier (Fig. 1). Furthermore, we observed that calcification within the fusiform mass and in the distal part of the deep posterior compartment had increased in comparison to that seen in the radiographs taken 10 years earlier. The erosion of the medial and posterior parts of the distal two-thirds of the fibula had also increased, and a sclerotic change in the lateral cortex of the middle third of the tibia was newly observed. We performed a computed tomography (CT) scan to examine the pattern and distribution of the calcifications more closely; peripherally distributed calcification was found in the anterior compartment and in the distal part of the deep posterior compartment, and sclerotic changes were found in the tibial and fibular cortex (Fig. 2). There was evidence of increased uptake between the tibia and fibula in the delayed bone phase on 3 phases of bone scan, which was determined to be caused by calcification.

Laboratory investigations revealed that the erythrocyte sedimentation rate (ESR) was 69 mm/h and the C-reactive protein (CRP) level was 5.80 mg/dL with no fever. Serum calcium, phosphorous, and alkaline phosphatase levels were all within the normal limits.

**Figure 3:**
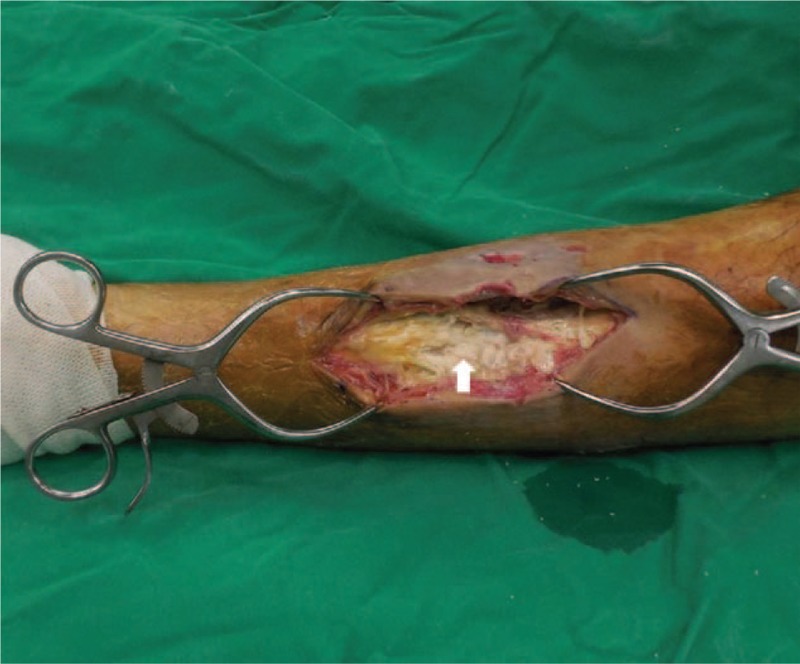
Intraoperative clinical photographs. The entire muscular portion of the tibialis anterior, extensor digitorum longus, and extensor hallucis longus was substituted with a whitish, toothpaste-like material (arrow).

**Figure 4:**
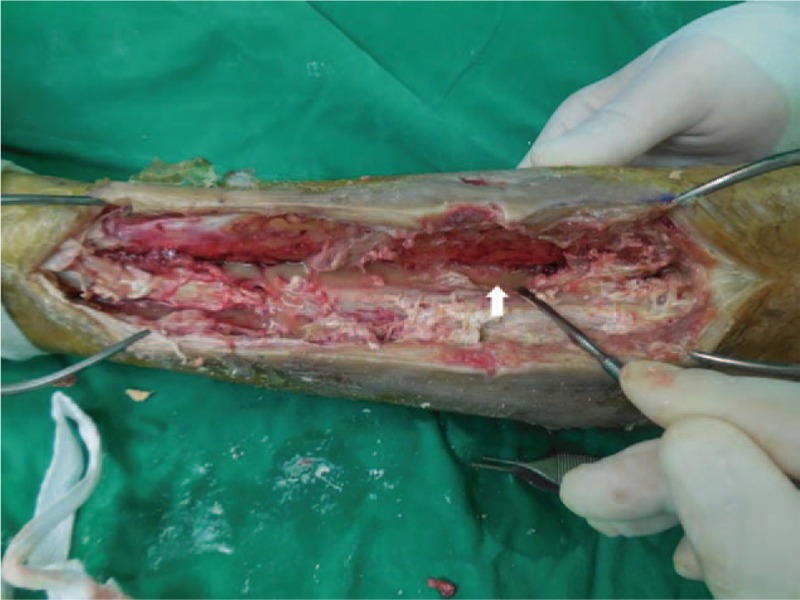
After *en bloc* resection of the anterior compartment, we approached through the interosseous membrane. As soon as we opened the interosseous membrane, turbid fluid flowed out (arrow).

**Figure 5:**
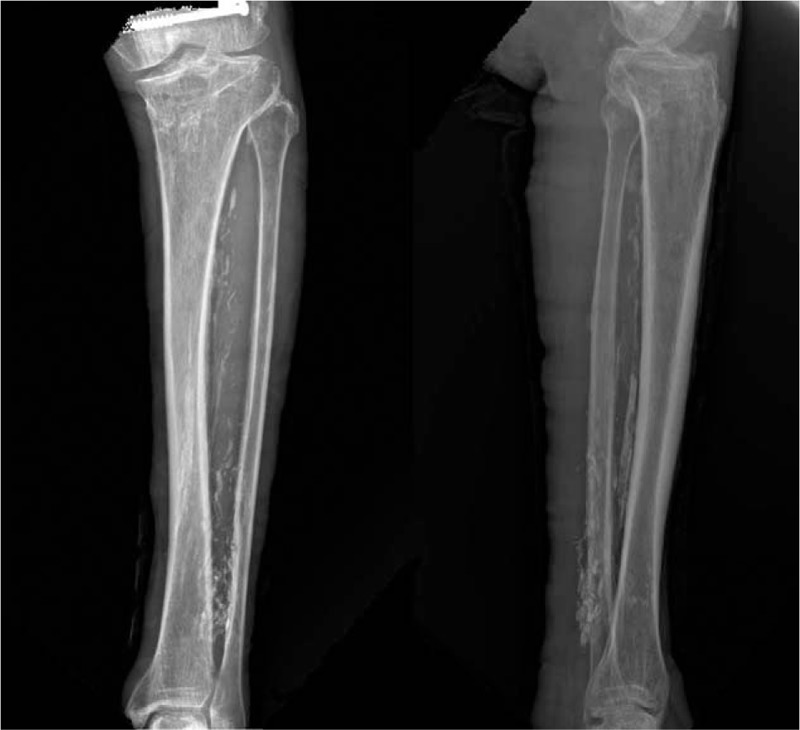
Postoperative radiographs of the left leg showing decreased radiopaque mass.

We performed an incisional biopsy to differentiated soft tissue sarcoma and no viable, malignant cells were found. A thick fluid with a chalk-like material continued to be discharged through the sinus opening. We performed an extensive debridement of the anterior and deep posterior compartments to ensure definitive treatment. Intraoperative findings showed that the deep fascia was thickened, and that the tibialis anterior, extensor hallucis longus, and extensor digitorum longus muscles had become necrotic and changed into a whitish, toothpaste-like material (Fig. 3). Hard calcific materials resembling rice grains were spread within the necrotic muscle. Furthermore, we observed an erosion of the cortex without intramedullary involvement on the lateral tibial surface and medial fibular surface. After an en-block resection of the anterior compartment, we approached the posterior compartment through the interosseous membrane. When we opened the interosseous membrane, the turbid fluid was drained, and we found that the flexor hallucis, flexor digitorum, and tibialis posterior muscles formed a twig-shaped, hard calcification in the direction of the muscle fibers, unlike in the anterior compartment (Fig. 4). Upon performing extensive debridement, we inserted a drain tube and performed primary closure. As *Pseudomonas aeruginosa* was identified in the intraoperative culture, we used antibiotic treatment (piperacillin-tazobactam, intravenously for 6 weeks and then ciprofloxacin, orally for 4 weeks). The fluid continued to be discharged through the drain even after the surgery; delayed wound healing occurred four weeks following the surgery, and there was no recurrence and the patients were satisfied with the result of the operation at follow-up conducted 2 years later (Fig. 5).

Pathologic evaluation revealed that the anterior compartment specimen had acute and chronic inflammation with dystrophic calcification, and the posterior compartment specimen had fibrosis with dystrophic calcification; the tibial periosteum had acute and chronic inflammation with dystrophic calcification, and the tibial bone was fibrotic. These findings led to the diagnosis of calcific myonecrosis.

## 3 Discussion

The symptoms of calcific myonecrosis appear, on average, 37 years following the initial injury (range: 10–64 years),^[5]^ and a typical finding is a firm or soft enlarging mass. The patient in this study had an 18-year interval before the onset of symptoms. At the time of the initial injury, the patient had a typical history of calcific myonecrosis, including compartment syndrome and sciatic nerve injury. However, he did not have the typical symptoms of calcific myonecrosis at the time of his visit to the hospital; instead, there was evidence of infection, including a sensation of warmth, redness, and elevated ESR and CRP. A spontaneous sinus was formed in a relatively short period of time after the onset of the symptoms. We cultured the patient's sample after the sinus tract was formed; it is not clear whether the calcified mass was infected via the sinus tract or was infected de novo. Nevertheless, we discovered that a calcified mass is susceptible to infection; when it is infected, antibiotic treatment via the bloodstream is insufficient because of its avascularity, and an extensive debridement is required.

The pathophysiology of calcific myonecrosis has not yet been elucidated. Although there was reported case of calcific myonecrosis in patient without previous trauma such as juvenile dermatomyositis,^[6]^ most cases of calcific myonecrosis involve high energy trauma.^[1,2,4]^ The central event is the loss of calcium regulation in the mitochondria owing to cell death.^[7]^ It is known that calcium begins to be deposited once its regulation is impaired; the deposited mass is enlarged by repeated intralesional hemorrhage and finally herniates through the muscle fascia due to focal enlargement.^[3]^

The natural course of calcific myonecrosis is unclear, as the symptoms manifest decades following the initial injury. In general, however, calcific myonecrosis is considered to be a benign entity. Muramatsu et al^[4]^ reported that a patient with calcific myonecrosis in the leg was seen to remain unchanged at 10-year follow-up. Alternatively, other studies suggest that calcific myonecrosis may also have an invasive appearance. O’Keefe et al^[3]^ reported the cases of three patients with calcific myonecrosis; 1 patient had extensive erosion of the fibula with chronic reactive periostitis and cortical thickening of the tibia, whereas the other 2 patients had smooth erosion of the tibia. Zohman et al^[8]^ reported a case of calcific myonecrosis in which the calcification in the leg became larger, and extensive erosion of the fibula and a small erosion of the tibia was found at the patient's 7-year follow-up.

Similarly, we also observed increased calcification and erosion of the fibula and tibia without any intramedullary involvement at the 10-year follow-up of our patient. As shown in these cases, calcific myonecrosis sometimes has invasive features, and bone involvement is thought to be because of a chronic pressure effect. In particular, when there is extensive bone erosion, physicians must consider a differential diagnosis to rule out other malignant lesions that form calcifications, such as epithelioid sarcoma, synovial sarcoma, and soft tissue osteosarcoma.^[9]^

Although aggressive debridement and definitive soft tissue coverage are definitive treatments for calcific myonecrosis, surgical treatment comes with a high risk of complications. Viau et al^[10]^ incised, drained, and performed open treatment of the wound in 2 patients with calcific myonecrosis; in both cases, secondary infection occurred, and 1 patient had to undergo an amputation, whereas the other patient underwent chronic intermittent drainage. In 2006, O’Dwyer et al^[9]^ proposed that calcific myonecrosis should be considered a “don’t-touch” lesion, recommending simple observation for patients with asymptomatic calcific myonecrosis, and a complete surgical excision when a biopsy is required. Rynders et al^[1]^ suggested that the surgical indications of calcific myonecrosis are an infection or a draining sinus requiring incision and drainage; an uncertain diagnosis of calcific myonecrosis that requires ruling out malignancy; symptoms causing functional impairment in the patient; and cases where the patient desires improvement. We agree with these surgical indications. A limited surgery, such as the drain insertion performed for our patient, is not conducive to healing sinuses, and an extensive debridement should be performed when surgeries are required.

## 4 Conclusion

Calcific myonecrosis is a late sequela of trauma, such as with compartment syndrome, that induces vascular compromise. Calcific myonecrosis is mostly a benign entity, but some cases of calcific myonecrosis cause bone destruction and form nonhealing chronic sinuses. In such cases, surgical treatment is required, during which the necrotic tissue and calcific material must be extensively debrided and drained.

## Author contributions

**Conceptualization:** Duke Whan Chung.

**Data curation:** Jong Hun Baek.

**Investigation:** Jong Hun Baek.

**Supervision:** Bi O Jeong, Duke Whan Chung.

**Writing – original draft:** Jong Hun Baek.

**Writing – review & editing:** Bi O Jeong.

Jong Hun Baek orcid: 0000-0001-8631-9570
